# Pre-clinical Investigations of Therapeutic Markers Associated with Acute and Chronic Restraint Stress: A Nuclear Magnetic Resonance Based Contrast Metabolic Approach

**DOI:** 10.7150/ntno.76294

**Published:** 2023-01-01

**Authors:** Sanjay Singh, Sukanya Tripathy, Atul Rawat, Durgesh Dubey, Sarfraj Ahmad Siddiqui, Rajesh Ugale, Dinesh Kumar, Anand Prakash

**Affiliations:** 1 Department of Biotechnology, Babasaheb Bhimrao Ambedkar University, Lucknow, India.; 2 Centre of Biomedical Research, Lucknow, India.; 3 Department of Biotechnology, Mahatma Gandhi Central University, Bihar, India.; 4 Department of Pharmaceutical Sciences, Rashtrasant Tukadoji Maharaj Nagpur University, Nagpur, India.

**Keywords:** Metabolomics, NMR, Metabolic Patterns, Restraint Stress, Behavioural Metabolomics

## Abstract

Stress can be defined by two parameters, first the psychological sensing of pressure and second is the body's response. However, the exposure time to stress depicts the biological response produced against it. The effect of acute and chronic restraint stress on anxiety and the production of systemic metabolites were investigated in male Sprague-Dawley (SD) rats. Behavioural test was performed on elevated plus maze (EPM) in conjunction with the statistical analysis that exhibited the habituation during long term exposure to stress when compared with the short-term stress. These behaviour-based changes resulted in interpolated concentration of some serum metabolites like carbohydrates, amino acids and lipids as analysed by NMR. Metabolic analysis along with the multivariate analysis demonstrated that the expression of concentration of metabolites including glutamate, proline, succinate, citrate, and tyrosine is higher in the acute stress than the chronic stress, while glucose and lipids i.e., LDL and VLDL changed in the opposite trends. Thus, the aforesaid study provides an analytical strategy for the characterization of perturbed metabolites induced due to the behavioural modifications in an organism. It may further aid in developing potential therapeutic markers at the metabolic levels which may broaden the treatment options for stress and anxiety related disorders.

## Introduction

The term “stress” is defined as any sort of pressure that gets an organism's defences up and threatens its existence. The sensed threat to an organism is referred to as “stressor” and the reaction produced to it as the “stress response.” Based on the assessment of perceived threat the organism develops the coping responses 1. It was found through studies correlating stress and behaviour that on the basis of timing of the exposure, stressor is of two types: acute and chronic. Acute stress suggests that the exposure to the stressor is for a shorter duration whereas chronic stress persists for a longer duration 2. During stress the homeostasis of an individual gets altered 3 and as thermodynamic law suggests “everybody in the universe tends to be in the state of stability”, the stressed individual also shows some adaptive behaviour to habituate with the long duration stress i.e., chronic stress 4. On the other hand, acute stress due to its short term and sudden exposure may leads to the anxiety like conditions 5. The kinship between psychological stressors and chronic disease is complex and is affected by the quantity i.e., number of times the stressor is exposed and duration of stressors.

The most suitable method to develop a model for stress in laboratory is restraint stress. Due to the fact that restraining the movement is relatively an easier way to induce stress, many researchers have used restraint in order to examine the effects of stress on learning, memory and anxiety 6. Restraint is considered a psychological stressor for animals. It does not physically harm the animal, rather restrict the movement of the model for a defined period of time and only breathing is allowed. As with many stressors, intensity and timing of restraint stress strongly influence learning in animals. The exposure of a stressor for a shorter duration (6 hour, 1 day) is considered as acute stress and if it is for 6-7 hr for one week then it is chronic stress 7. On further analysis at molecular level, it was revealed that such type of stress activates the HPA-axis and increases the production of glucocorticoids 8. Although the varying type of stress produces the cumulative response rather than the individual response for each type of stress 9. It was revealed through exhaustive studies that during stress, hormones and metabolic profiles of an organism gets altered 10. Thus, the metabolic profiling will prove to be a valuable aid to provide insight into physiology of stress response and developing a putative cure for such behavioural paradigms.

Metabolomics refers to the systematic evaluation of small molecules known as metabolites within cells, body fluids or tissues which provide insights of metabolic perturbations in biological systems and is routinely used to evaluate systemic responses to any subtle pathophysiological stimuli or stress. It also provides opportunities for developing diagnostic/prognostic biomarkers related to the disease or a specific pathophysiological condition. Over time, it has been accepted as a valuable complementary approach to genomics, transcriptomics and proteomics to provide a complete understanding of the disease mechanism. Metabolomics approach relies on the data obtained by using high throughput analytical techniques like Nuclear Magnetic Resonance (NMR), Mass Spectrometry (MS), High Performance Liquid Chromatography (HPLC) and Gas Chromatography (GC) followed by multivariate data analysis. The NMR based metabolic profiling in conjunction with multivariate data analysis is an ideal platform and has been widely used to explore metabolic changes in biofluids (serum, plasma, urine, CSF, etc.) for mechanistic study of diseases or potential biomarker discovery, owing to applicability to a variety of sample types, non-destructive nature, rapid collection of data, reproducibility, quantitative and no tedious sample preparation procedures 11.

## Materials and Methods

### Animal model

The procedures related to maintenance and experimentation were carried out in accordance with the ethics guidelines laid for animal administration and approved by the Animal Care and Use Committee (IAEC/UDPS/2016/40). A total of 30-35 male Sprague-Dawley rats which were 80-90 days old and weighed 100±20 grams were used in the experiment. Animals were maintained in a temperature-controlled room (25±2 °C) and were housed individually in a separate cage on a 12 hours' light/dark cycle (lights on at 7:00 a.m) with free access to food and water. Prior to the onset of the experiment, animals were handled for 3-5 minutes daily for two weeks to make them accustomed to being handled. All animals were kept in their home cages and were randomly assigned into the following three groups- normal control (NC), acute stress (AS), and chronic stress group (CS), each group has 10 animals. All animals were kept under strict observation for the next 7 days and weighed each day. The protocol used in the study is summarised in the Supplementary Figure S1A.

### Restraint Stress Procedure

The restraint stress protocol was followed as Derek, 2011. Animals were wrapped in a plastic bags customised in the form of a cone in such a manner that the animal becomes completely immobilized till the end of experiment but only be able to respire comfortably by an opening created at the rostral end of the animal and they were not allowed to access either food or water during restraint stress period (Supplementary Figure S1B). Chronic restraint stress was given for 7 days (6 h/day). For acute restraint stress, rats were subjected to the stress for 1 day for 6 h. Control animals were not subjected to any type of stress. After the offset of the stressful period, blood serum was isolated from half of the animals and the remaining half proceeded for the EPM (Elevated plus maze) test.

### Elevated Plus Maze (EPM)

The EPM apparatus consists of four arms opposing each other at the same distance with an angle of 90° Supplementary Figure S1C. All the four arms were composed of black coloured plexi-glass in such a way that two opposing arms remained open i.e. exposed to light whereas the other two remaining arms were closed (dark). Open arm had the dimension of 50 cm x 10.2 cm whereas the closed arm had the dimension of 50 cm x 10.2 cm x 40.6 cm. A camera was placed over the apparatus to record the entry and time spent in each arm by animals. The apparatus was positioned 60 cm above from the floor. Prior to the onset of EPM test, animals were allowed to habituate the EPM testing room for 1 h followed by placing the animals at the centre of arms facing towards closed arm and were allowed to move freely on EPM for 5 minutes. The open arm entries and percent time spent in open arm was calculated with respect to total number of entries and total time spent of 5 minutes respectively.

Percent time spent in open arm was calculated by a formula:



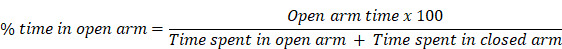



### Blood Samples

Following the termination of the restraint stress experiment on 7^th^ day, blood samples were isolated from each of the CS, AS and NC group animals in sterile 2 ml centrifuge tubes and were left to coagulate for 30 minutes at room temperature (37 °C). Next the tubes were centrifuged at 5000 rpm for 5 minutes at 4 °C, the pale yellow colour supernatant (serum) was collected in a new sterile centrifuge tube. The obtained serum was immediately kept at -80 °C until analysed by NMR.

### Sample Preparation

The serum samples were thawed and centrifuged at 10,000 rpm for 5 minutes to remove precipitates if any before sample preparation for NMR data acquisition. Sample was prepared as follows- 200 µl of serum sample was mixed with an equal volume of sodium phosphate buffer 20 mM and pH 7.4 with 0.9% saline prepared in D_2_O were mixed 12. A total 400 µl of sample was then transferred in to a 5 mm NMR tubes (Wilmad Glass, USA) for data acquisition, with a co-axial insert containing the known concentration of TSP (Sodium salt of 3-trimethylsilyl-(2,2,3,3-d4)-propionic acid) i.e. 0.1mM was used as external standard reference to aid metabolite quantification for NMR experiment. Deuterium oxide (D_2_O; as a co-solvent and to provide a deuterium field or frequency lock and the chemical shift reference (δ0.0) and sodium salt of trimethylsilyl propionic acid-d4 (TSP) used for NMR experiments were purchased from Sigma-Aldrich (Rhode Island, USA).

### NMR Experiments

All the NMR spectra were recorded on an 800 MHz NMR spectrometer from Bruker Biospin Avance at 300 Kelvin. Operating at a proton frequency of 800.21 MHz, equipped with CryoProbe and an actively shielded gradient unit with a maximum gradient-strength output of 53 G/cm. The acquired raw NMR data were processed in Topspin-2.1 (NMR data Processing Software) from BrukerBiopspin. On each of the serum samples, 1D ^1^H NMR spectra was recorded: transverse relaxation-edited CPMG (Carr-Purcell-Meiboom-Gill) spectra. The 1D ^1^H CPMG NMR spectra were recorded using the standard Bruker's pulse program library sequence (cpmgpr1d) with pre-saturation of the water peak through irradiating it continuously during the recycle delay (RD) of 5 sec. Each spectrum consisted of the accumulation of 128 scans and lasted for approximately 15 minutes. To remove broad signals from triglycerides, proteins, cholesterols, and phospholipids, a total spin-spin relaxation time of 60 ms (n=300 and 2T=200μs) was applied. Each FID (free induction decay) was zero-filled and Fourier-transformed to 64 K data points following manual phase and baseline-correction using Bruker NMR data Processing Software Topspin-V3.5. A line broadening factor of 0.3 Hz and a sine-bell apodisation function was applied to FIDs before Fourier Transformation. After FT, the chemical shifts were referenced internally to the methyl peak of L-Alanine (at δ=1.46 ppm).

### NMR Data Processing and Spectral Assignment

All the ^1^H NMR spectra acquired on the serum samples were manually phase and baseline -corrected, and further an overlay of all the spectra was visually inspected for their acceptability for binning or bucketing. The spectra were then integrated into regions with a bucket width of 0.01 ppm using the AMIX package (V3.8, BrukerBiospin, Germany) in the spectral region (δ0.5-8.5 ppm). To eliminate the effects of imperfect water suppression, the region of (δ4.7-5.0) ppm for the water signal was discarded in the serum spectra. The binned data is finally subjected to multivariate statistical analysis using the open access web-based metabolomic data processing tool, named MetaboAnalyst to identify the altered metabolic pattern.

Spectral assignment is a prerequisite for multivariate analysis. Hence processed (phase and baseline corrected) representative spectra from each group was selected for assignment. The 1D ^1^H CPMG NMR spectra metabolite resonances were assigned as far as possible using the Chenomx NMR suite (Chenomx Inc., Edmonton, AB, Canada) 13. The remaining peaks in the CPMG ^1^H NMR spectra were assigned using previously reported NMR assignments of metabolites 12, 14-15, data obtained from BMRB database (Biological Magnetic Resonance Data Bank), HMDB (The Human Metabolome Database) 16 and MMCD (Madison Metabolomics Consortium Database).

### Multivariate Data Analysis

The binned data were collected into Excel (Microsoft office 2010), and further used for univariate and multivariate analysis using the open access web-based metabolomic data processing tool, named MetaboAnalyst 3.0 17. In the MetaboAnalyst, data processing was done: using the default practice for data integrity check and missing value estimation; data filtering using interquartile range (IQR) to identify and remove variables that are unlikely to be of use when modelling the data; scaling method Pareto was used. After data processing and normalization, the data was subjected to multivariate statistical analyses methods, i.e. unsupervised principal component analysis, (PCA) and supervised partial least squares- discriminant analysis (PLS-DA). PCA is used to detect intrinsic clusters and outliers within the data set, while PLS-DA maximizes class discrimination. To assure the robustness of the model and to avoid the over fitting of the PLS model, the cross-validation (CV) method- “10-fold CV” algorithm has been used for model validation, from which using the top latent variables, a 100% classification accuracy and R2 and Q2 values were extracted. R2 represents the variance captured by the model and Q2 represents the prediction ability of the model, based on the goodness of fit parameter and goodness of prediction parameter respectively. Interpretation of the PLS-DA model was based on the score plot, regression coefficients and the variable importance in the projection plot (VIP). Significantly altered metabolite entities were identified based on their significantly higher values of VIP scores and coefficient values. The coefficient importance is based on the weighted sum of PLS-regression scores; whereas, the VIP score represents a weighted sum of squares of the PLS loadings and indicate the importance of the variable to the whole model and the corresponding coefficient values attribute its discriminatory potential. Generally, the variables (or metabolite peaks) with high VIP and coefficient scores indicate that it is important for class discrimination. The box plot representation (evaluated through univariate analysis) was used to visualize the variation in the levels of significantly altered metabolites identified in the multivariate analysis. Further validation was performed using the hierarchical cluster analysis (HCA) methods. HCA algorithm is a commonly used unsupervised clustering method. Agglomerative hierarchical clustering begins with each sample as a separate cluster and then proceeds to combine them until all samples belong to one cluster. Spearman distance measure and a clustering method Ward's linkage was used to produce a dendrogram to evaluate the overall similarity/dissimilarity between the normal control and stressed (acute or chronic) animals.

## Results

### Elevated Plus Maze Experiment

**Figure 1:**
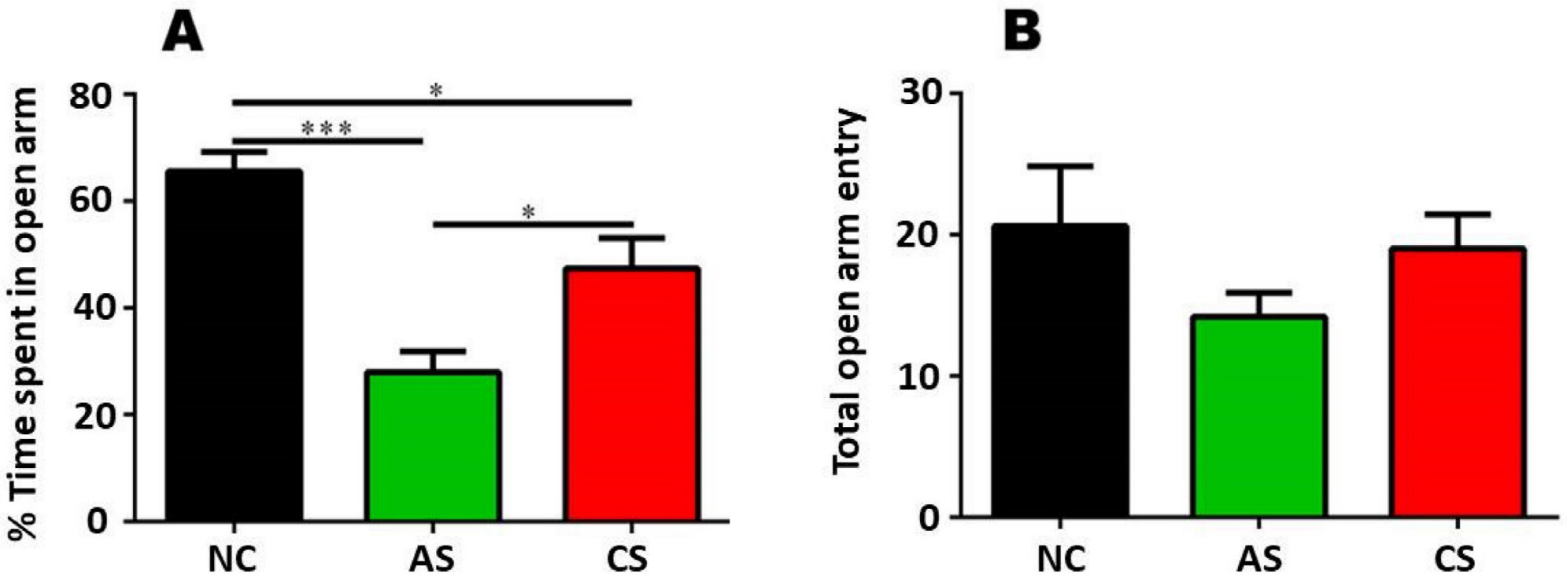
** (A)** is showing the % time spent in open arm following AS and CS restraint stress exposure as compared to the NC group **(B)** exhibits no significant change across the group in terms of total open arm entries as exhibited by AS, CS and NC group.

We used the EPM test to know the effect of stress duration exposure, i.e. acute and chronic restraint stress, on anxiolytic or anxiogenic behaviour of animals. Usually, the animals have a tendency to avoid open arms and prefer closed arms to reside. Repeated entry and time spent (calculated in %) by the animal in the open arm represents anxiolytic behaviour while anxiogenic behaviour is governed by the repeated entry into the closed arm and decreased exploration of the open arm (Figure 1).

We observed that % time spent in the open arm following acute stress (AS) and chronic stress (CS), AS grouped animals exhibited increased level of anxiogenic response (decreased % time spent in open arm) as compared to the NC group as well as from the CS group. We further confirmed these changes via one-way ANOVA analysis that revealed, AS group and CS group exhibited decreased level of % time spent in open arm as (anxiogenic response) compared to that of NC group [F (2, 12) = 17.39]. One-way ANOVA analysis followed by Tukey's multiple post-hoc comparison depicted that AS group (*p*<0.001) and CS group (*p*<0.05) have increased anxiogenic response when compared with the NC group. Further, changes were also found to be significant between AS and CS group (*p*<0.05) [F (2, 12) = 17.39] which proved that AS group presented more anxiogenic behaviour than the CS and control group.

Along with the % time spent in open arm, number of entries in open arm was also counted that depicted no change across the groups. The observed data was analysed by One-way ANOVA that confirmed there was no significant difference across the group when compared with the NC group [F (2, 12) = 1.242, p>0.05]. These changes were further confirmed by Tukey's post hoc multiple comparison analysis that revealed AS group (p>0.05) and CS group (p>0.05) exhibited insignificant changes as compared with the control group [F (2, 12) = 1.242, p>0.05].

### Effect of Acute stress and chronic stress on the body weight of animals

Our next aim was to observe the effect of AS and CS on the body weight of animals. We found that CS group showed a significant decrease in body weight while AS group showed no change in body weight as compared to the NC group. The changes were confirmed by the one-way ANOVA analysis that revealed insignificant changes in the AS group (p>0.05) but the changes were significant in the CS group (p<0.05) as compared to the NC group. Tukey's post hoc analysis further confirmed that the CS group exhibited decreased body weight as compared to NC group [F (2, 18) = 4.526, (p<0.05)] Supplementary Figure S2.

### Spectral Assignment

**Figure 2:**
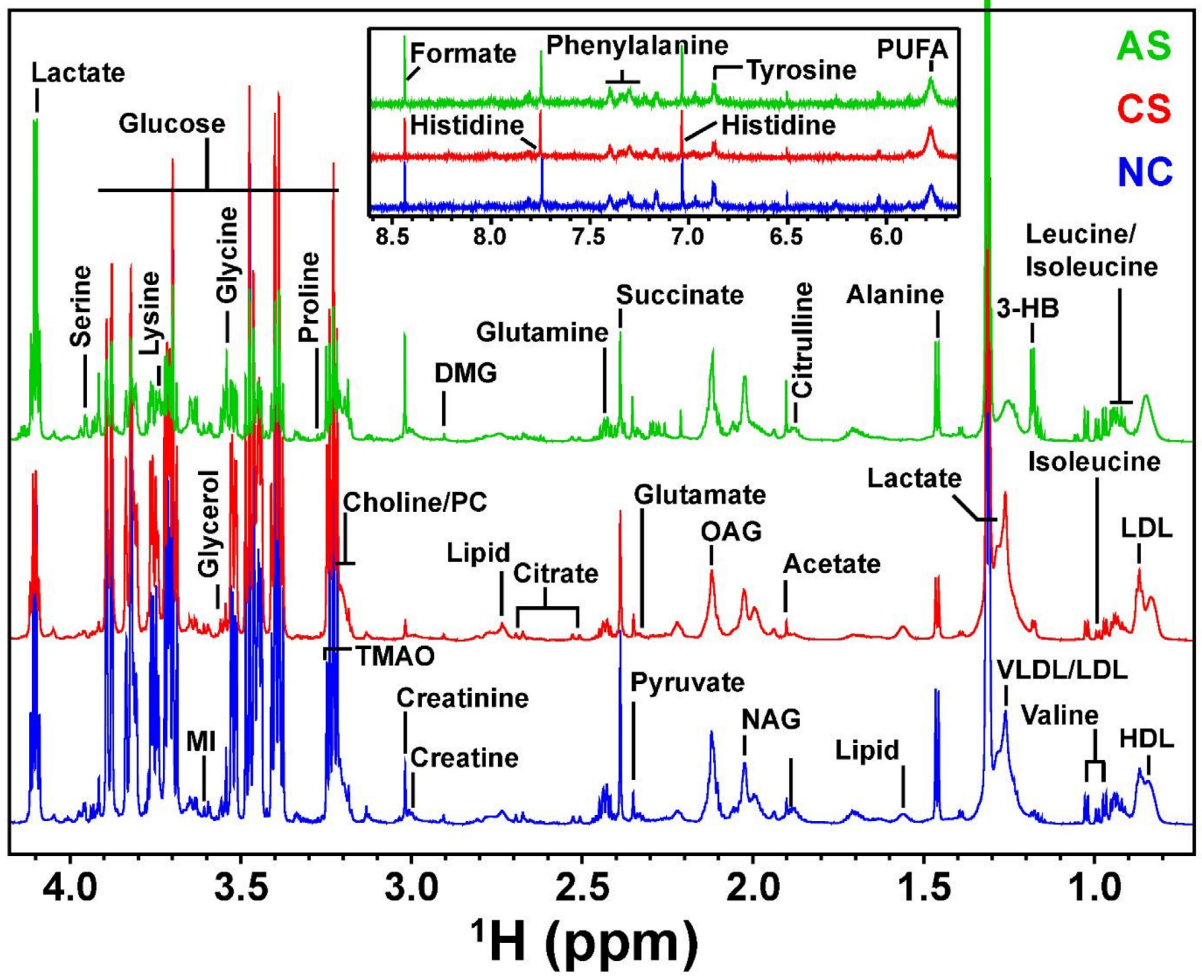
Stack plot of representatives 1D ^1^H CPMG NMR spectra of serum samples from each group NC, CS and AS acquired at 800 MHz.

The representative 1D ^1^H CPMG NMR spectra of rat serum samples obtained from different groups (NC, AS and CS) with the assigned resonances of relevant metabolites are shown in Figure 2. The serum spectra contained peaks mainly from lipoproteins (high-density lipoprotein [HDL], low-density lipoprotein [LDL], very low density lipoprotein [VLDL], polyunsaturated fatty acids [PUFA]) glycoproteins (N-acetyl glycoproteins [NAG], and O-acetyl glycoproteins [OAG]), amino acids (alanine, valine, lysine, leucine, isoleucine, phenylalanine, histidine, tyrosine, glutamine, glutamate, proline, etc.), glucose, lactate, pyruvate, succinate, creatine, creatinine, acetoacetate, acetate, and choline-containing metabolites.

### Multivariate statistical Analysis

**Figure 3:**
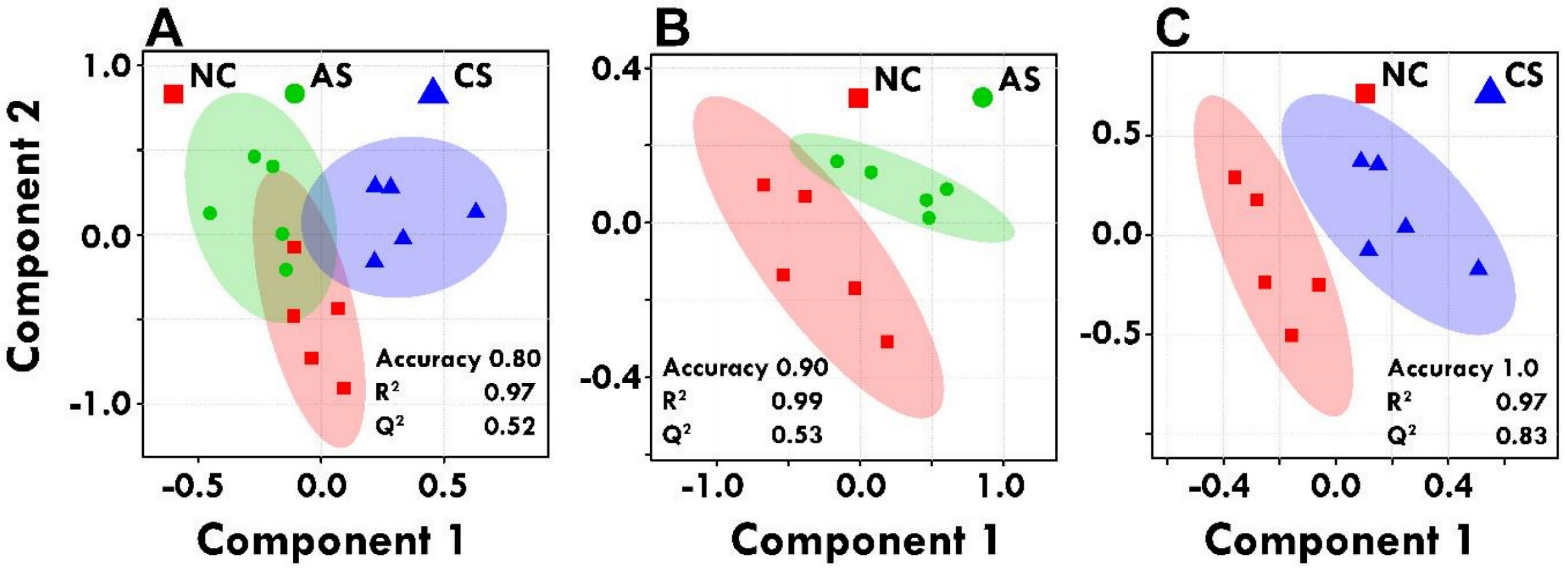
PLS-DA analyses were performed using MetaboAnalyst 3.0. Score plots of PLS-DA model among control and acute and chronic stress groups **(A)**; PLS-DA model between control and acute stress group **(B)**; PLS-DA model between control and chronic stress group **(C)**; along with their R^2^ and Q^2^ values. The shaded areas are the 95% confidence regions of each treatment.

A five-component principal component analysis (PCA) and partial least squares-discriminate analyses (PLS-DA) were applied to investigate patterns in variables arising from ^1^H-NMR spectra obtained from the serum samples. First, aiming to get an overview of the metabolite patterns of the groups an unsupervised multivariate data analysis was performed. PCA is an unsupervised pattern recognition tool that attempts to explicate the maximum amount of variance underlying in a multi-dimensional dataset. As such, PCA was applied to investigate patterns between ^1^H-NMR profiles within the groups. Additionally, PCA was used to screen for outlying samples, if the sample fell outside a 95% confidence interval region. The principle component analysis (PCA) model is given in Supplementary Figure S3. Next, a supervised model, partial least squares-discriminant analysis (PLSDA), was applied, Figure 3. PLS-DA revealed differences in the different groups, which is necessary to eliminate outliers and enhance the quality of the PCA model. PLS-DA tends to over fit the data, hence the model needs to be stringently validated to see if the separation is statistically significant or is due to random noise. To avoid the over-fitting of the PLS-DA model, 10-fold cross validation was carried out to measure the robustness of the PLS-DA model obtained because of the small number of samples. The quality of the PLS-DA model is described by the measures of Accuracy, R^2^ and Q^2^ values; which helps to evaluate 100% classification accuracy using the top 5 latent variables- R^2^ is defined as the proportion of variance in the data explained by the model, indicating goodness of fit, whereas Q^2^ is defined as the proportion of variance in the data predicted by the model, indicating predictability, respectively Figure 3.

**Figure 4:**
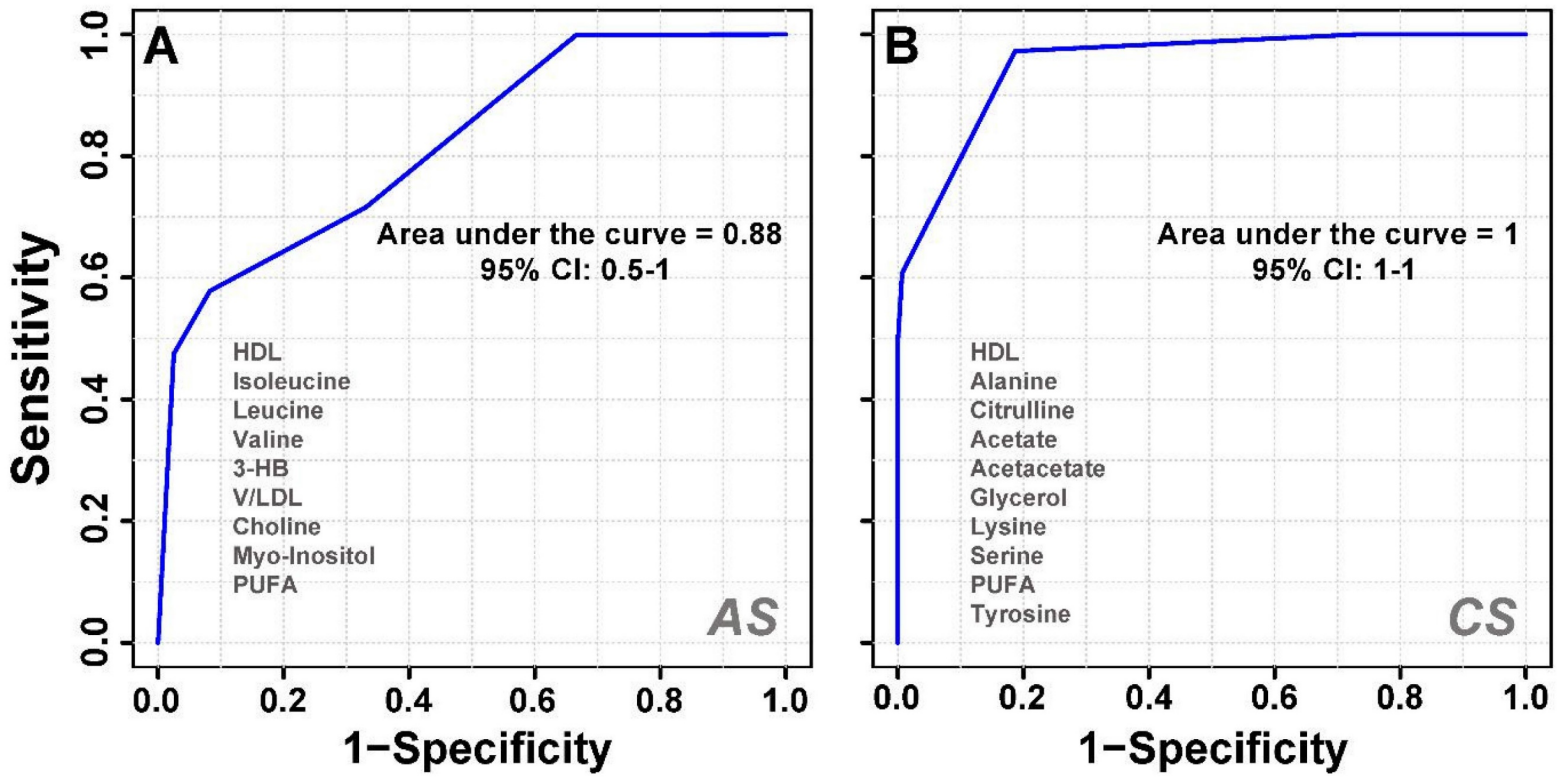
A cumulative AUROC (Area under the receiver-operating characteristic) curve of the significant metabolites with (AUC>0.9) that decreased or increased with respect to the control group. **(A)** Acute stress and **(B)** Chronic stress; derived from the CPMG ^1^H NMR spectra.

To determine significantly differential metabolites, metabolites were filtered based on the following criteria: (i) PLS-DA; VIP ≥ 1 and coefficient score ≥ 30 (ii) p value < 0.05. The identified significant metabolic entities are shown in Table 1. Further receiver's operating characteristic (ROC) curves analysis was also performed for the significant metabolite markers to evaluate their predictive power or diagnostic accuracy. The area under the ROC curve gives of discriminatory ability (0.5=no discrimination; 1=perfect discrimination). The largest and smallest resulting AUC values range from 1.0 to 0.6 (Table 1) which indicated that these metabolites could be potential biomarkers for diagnosis and surveillance for early stress markers. Representative ROC curves for some of the serum metabolites significantly altered in acute and chronic -stress are shown in Figure 4. The boxplot representation was used to visualize the variation in the levels of significantly altered metabolites in acute and chronic -stress identified in the multivariate analysis evaluated through one-way-ANOVA. A 0.05 level of probability was used as the criterion for statistical significance (Supplementary Figure S4).

Further, validation was performed using the unsupervised hierarchical clustering analysis (HCA) methods in MetaboAnalyst. As also evident from the heat map shown in which clearly shows that metabolic perturbation is visually distinguishable from the control group, which clearly separates the up-regulated (in red) metabolite entities from the down-regulated (cyan) metabolite entities. Heat map wasgenerated using Pearson's correlation based dissimilarity measures and a clustering method named Ward's linkage using only the significantly altered metabolite entities Supplementary Figure S5A. Spearman distance measure and a clustering method Ward's linkage was used to produce a dendrogram (Supplementary Figure S5B). The hierarchical clustering performance showed the overall similarity/dissimilarity in CPMG spectra of acute and chronic -stress compared to normal control.

### Metabolic Pathway Analysis

**Figure 5:**
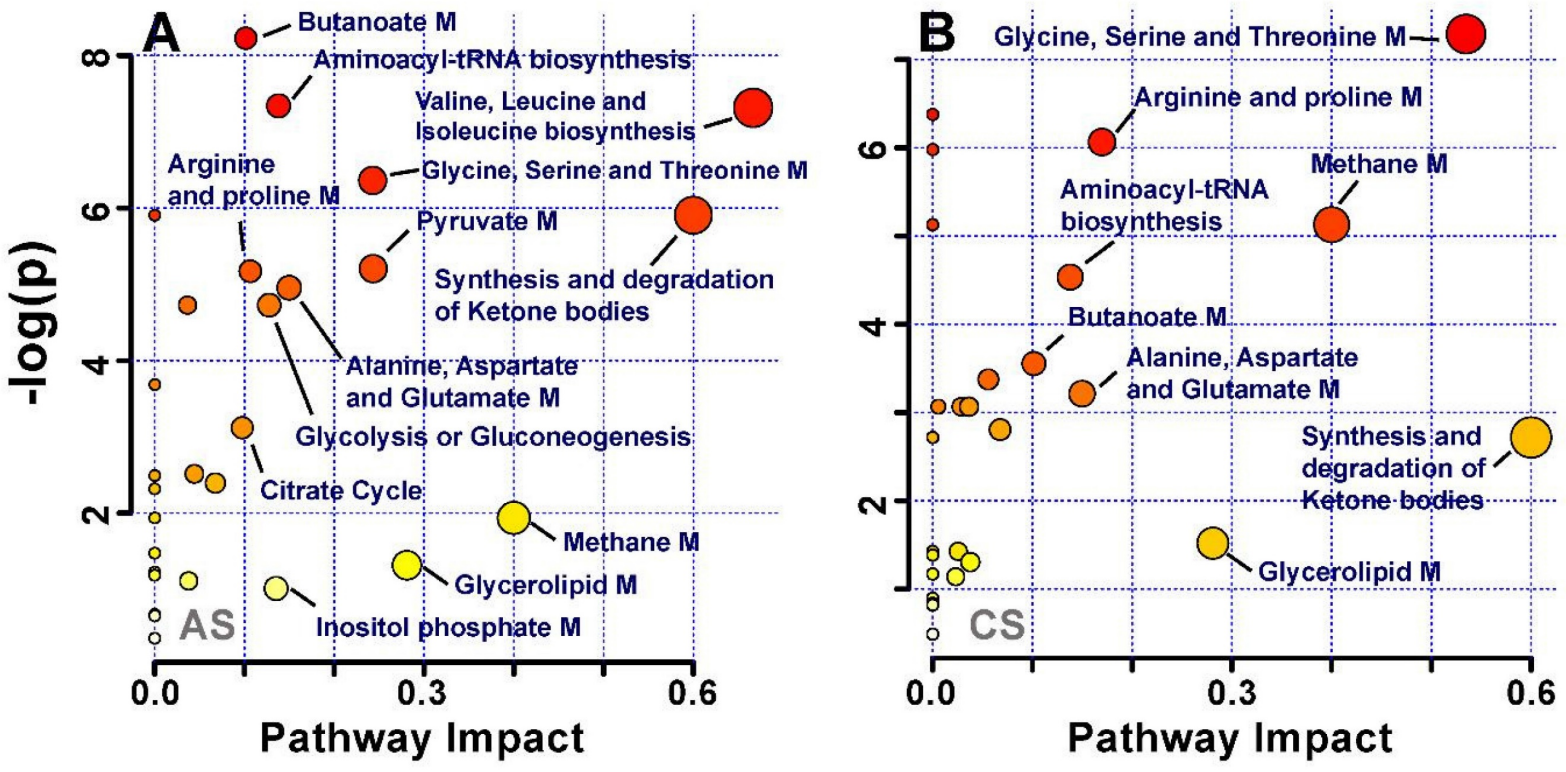
Pathway analysis of identified metabolites in the different groups after exposure to (**A**) acute stress and (**B**) chronic stress.

Multivariate pattern recognition analysis enabled us to identify the metabolites that significantly altered in acute and chronic -stress groups, (Table 1). These metabolic entities were subjected to pathways analysis, to identify the affected metabolic pathways and facilitate further biological interpretation. MetaboAnalyst supports pathway analysis (integrating enrichment analysis and pathway topology analysis). This web-based function implemented in MetaboAnalyst enables identification of altered metabolic pathways from its extensive KEGG database of pathways and metabolite libraries by simply uploading a list of compound names 18. The lipids and glycoproteins such as LDL, VLDL, HDL, N and O -acetyl glycoproteins were not recognized by the pathway analysis module; thus, were excluded from the analysis. The final list of altered metabolites was uploaded and analysed by using pathway library for Rattus norvegicus and pathway analysis algorithms; Over Representation Analysis: Hypergeometric Test and Pathway Topology Analysis: Relative-between Centrality in MetaboAnalyst. One-tailed p values are provided after adjusting for multiple testing. The output of this program will mark a metabolic pathway as significant if significantly more compounds involved in that pathway are present in the input list than would be expected by random chance. Figure 5 summarizes the pathway analysis, the perturbed metabolic pathways are mainly involved in glucose and energy metabolism, synthesis and degradation of amino acids, and lipid metabolism.

## Discussion

The present study demonstrated that the exposure to stressors differentially affects the behaviour of an organism. On analysing the stress-dependent anxiety in the EPM, we found that the CS group behaves more or less like the NC group whereas the AS group showed a decrease in % time spent in the open arm as compared to the control and chronic stress group. Similarly, a study by Derek 2011, revealed that there is a significant difference between the rats undergoing chronic stress and acute stress. It was found that the anxiety-like behaviour is more prominent in the rats which are exposed to stress for a shorter duration. Statistical analysis by one-way ANOVA revealed that duration and intensity of exposure significantly affects the time spent in open arm in organisms. The rats of the CS group spent more time in the open arm as compared to the AS group. When the number of entries in open arm was counted we found no significant differences among the groups. This can be attributed to the “habituation” of animals towards stressor more than those who underwent a one-day stress. Other probable reason for this behaviour is the lag between latter and former sessions of exposure to stress. A study performed, states that the anxiety-like behaviour is much more noticeable in the acute stress group than the chronic group 10. It was clearly depicted that very minute significant change was observed in one-day session stress but the 7-day stressed group spent more time in the open arm than the acute group.

The HPA axis exerts a negative feedback loop thus increasing the level of corticosterone. This enhanced level of cortisol directly affects the anxiety during stress. Along with the differential behaviour of stress groups on EPM, it was observed that the weight of an organism is also altered. Although maintaining the appropriate body weight is a remarkable sign of health and can prevent many diseases specially obesity. Usually, any kind of stress results in abnormal body weight and food intake suggesting the strong relation in between. Although several models to induce stress are available but among them the restraint stress is proved to be the most common as it can powerfully exerts the physiological and physical stress 36. However, there are studies that reported contrasting results during exposure to chronic stress on the food intake and body weight of animal 37,38. Recently Kuti et al., 2022 39 explored that acute exposure to stress have no significant alteration on the body weight while the animals underwent chronic stress showed decreased body weight when compared to the control group and these results are favouring our results. During the chronic stress, the activated HPA axis along with the serotonergic and catechlamine system have the ability to promote loss of appetite and can reduce the body weight 40. Similarly our results showed that AS group shows no change in body weight as compared to the naive group, while CS group shows a decrease in body weight on subsequent exposure to stress. The loss of weight is due to lesser appetite as compared to that of Control and AS group. The explanation to this also relies on the HPA activity. Gradual increase in serum corticosterone levels and decreased leptin levels following restraint attributes to lower body weight.

However, the current study conducted only on the male rats only and it is due to the behavioural alteration of male and female rats. Donner and Lowry, 2013**^41^** reported that during non-stressful behavioural tasks female rats exhibited less anxiety behaviour than males but during chronic stressful condition males exhibited consistent increase in anxiety while female's response was diverse and it depends upon the behavioural task applied, stressor and importantly oestrous cycle**^42^**. During oestrous cycle in females, the secretion of two major hormones oestrogen and progesterone fluctuates effectively. As these sex hormones are lipophilic in nature and has the ability to cross the blood brain barrier (BBB) and thus can alter the behavioural response. Indeed in the rodents, the estrous cycle lasts for 4 days and there are 4 phases of this cycle serially proestrus, estrus, metestrus and diestrus; Gouveia Jr et al., 2004**^43^** reported that during the diestrus phase female rats exhibited comparatively high anxiety like behaviour in T maze than the male rats. So, to avoid such fluctuation in behaviour, we used only male rats instead of females.

The present study has used ^1^H NMR based metabolomics approach to visualize the metabolic changes in the AS and CS restraint stress rat model. As observed the animals presented with restraint stress, AS or CS showed anxiety-like behaviour which prompted us to study the metabolic changes in these animals. Previous literature reported increased level of Glucose and cortisol but no change in the triglyceride level in AS group in comparison with the control group. However, the CS resulted in the decreased level of triglyceride and increased level of cortisol but no change in the glucose level as compare with the NC grouped animals 39. Besides these biomarkers both the stress group showed decreased respiratory exchange ratio 39. However, our study reported the key metabolites chiefly responsible for discrimination between the AS and CS groups are energy metabolites (glucose and amino acids) as well as membrane constituents (lipids and fatty acids). The lipids and amino acids are -constituents or itself- are neurotransmitters and very much responsible for normal functioning of both physical and psychological functions including muscles, glands, heart rate, appetite, sleep, behaviour and memory. Hence the increased or decreased levels of the metabolites (neurotransmitters) play a vital role in physical and psychological functioning of an organism in stress. There has been increasing evidence for the adverse effects of stress on human health, that include respiratory, gastrointestinal and cardiovascular -disorder, diabetes, and the immune system. Keeping this in mind the altered level of metabolites and their role in stress physiology has been discussed further. The increased and decreased level of metabolites in AS and CS are shown in Table 1. The AS groups were found to have the depleted levels of V/LDL, suggesting enhanced β-oxidation required to maintain the production of lipid and membrane metabolites and energy homeostasis in conditions of acute stress and starvation. The antioxidant and anti-inflammatory properties of HDL may be attributed to their increased levels in serum 20. In these conditions, an increase in the use of ketone bodies as a source of energy, consistent with the higher observed levels of acetate, 3-HB and acetoacetate in blood clearly indicates the active lipid metabolism. Whereas the CS groups were found to have elevated levels of lipids HDL, LDL, and VLDL along with a decreased level of acetate, 3-HB and acetoacetate suggesting an inhibited lipid metabolism. The decrease in the serum, ketone bodies, indicates a reduction of energy production through fatty acid oxidation, which is proven by increased levels of lipid in the serum. Further, glycerol, phosphocholine and myo-inositol, have an impeccable role in the synthesis of membrane phospholipids and lipid metabolism**^21^** in the cell, were found to be perturbed in the AS and CS animals. Choline, an important constituent of cell membrane and phospholipid metabolism are breakdown products of phosphatidylcholine. The rise in serum choline and phosphocholine concentrations in AS, together with a decreased serum level of lipids and lipoproteins, demonstrate the disruption of the cell membrane fluidity, possibly leading to enhanced membrane permeability and altered membrane structure caused by lipid peroxidation 22. Contrary to this the CS groups were found to have depleted levels of choline and phosphocholine. The depleted phosphocholine may reflect an enhanced degradation of cellular membranes in the brain, hitting the disruption in membrane transportation or barrier function. Choline is a precursor of acetylcholine, the decreased levels of acetylcholine has varied effects from anxiety, memory and learning disturbance to Alzheimer's disease 23-24. Unsaturated fatty acids, which are important in maintaining the relative fluidity of cell membranes and normal cell function, are released into circulation under stress, damaging the stability of cell membranes.

Glucose, a primary energy substrate for brain metabolism, plays an important role in energy homeostasis. Glucose levels were found to be significantly depleted in AS group, as compared to the controls. Increased metabolism of glucose in concurrence with up-regulation of pyruvate, along with the decreased level of succinate (intermediate of the TCA cycle), in the serum might be attributed to energy metabolism alterations of the TCA cycle and glycolysis 25. At the same time, the increase in serum lactate, the end-product of glycolysis, indicates the inhibition of glycolysis and gluconeogenesis. This may be related to anaerobic cell respiration, an energy metabolism process that indicates low metabolism efficiency. The reduced activity of the TCA cycle could reduce ATP generation in mitochondria and potentially induce fatigue, a symptom frequently observed in depressed patients and animals. These changes suggest that dysfunction of carbohydrate and energy metabolism may be present in stressed animals. The increased level of glucose to that of decreased level of lacate, the endpoint product of glycolysis pathway- suggests impaired glucose uptake and attenuated glycolysis in CS animals. A similar high glucose and decreased levels of lactate has also been reported in rat model of Huntington disease 26.

Amino acid metabolism is staggeringly complex since large numbers of metabolites and multiple metabolic pathways are involved. The perturbation in amino acids levels contemplates cellular demands for higher turnover of structural proteins in maintaining energy homeostasis probably induced by gluconeogenesis, under hypoxic conditions 27. Under oxidative stress the cellular metabolism switches from aerobic to anaerobic, to carry out the vital cellular processes. The lipids and carbohydrates can't be utilized as an energy source due to limited supply of oxygenated blood in a hypoxic environment, the reliance on alternative energy substrates (amino acids) increases which can be oxidized anaerobically with lower contribution to acidosis. As amino acids act as direct source of substrate for energy production and are an important constituent of glucose metabolism, their utilization is considered to alter according to the changes of carbohydrate or lipid utilization. The levels of glucogenic amino acids (GAA) (glutamate, glutamine, and serine) and ketogenic amino acids (leucine and lysine) were found to be elevated in AS groups along with the branch chain amino acids (BCA's). The BCA's are catabolized to replenish the depleted levels of TCA cycle intermediates or as acetyl derivatives to generate energy during stress suggesting dampened glycolysis alongside muscle and protein breakdown. Not only this, they are also equally important for protein biosynthesis as well as biosynthesis of several biogenic amines important for survival in conditions of acute stress 28. GAA's are considered to be very important for maintenance and promotion of cell function. They are also an important part for excretion of the nitrogenous waste and key energy metabolites to be metabolised in oxidative stress. Citrulline is found to be upregulated in inflammation or oxidative stress. The levels of glucogenic (glutamate, glutamine, alanine, and serine) and ketogenic amino acids (leucine and lysine) were found to be dampened in CS animals. The metabolites glutamate, glutamine, glycine and serine are foundto involved in a number of cellular processes in the mammalian brain 29. The glutamate-glutamine cycling and its connections to brain biosynthesis from glucose of glutamate and their subsequent metabolism not only work as energy-providing materials, many, including GABA, glycine, etc., also serve as functional neurotransmitters. Glutamate is the primary excitatory neurotransmitter 30. Glycine is an inhibitory neurotransmitter 31. The decreased levels of several amino acids in the sera, suggests aberrant amino acid catabolism and protein biosynthesis. Glutamate and glutamine are both precursors for two particularly important antioxidants, glutathione and taurine.

An increased level of creatine and creatinine was found in the serum of AS animals in comparison to control. Creatine/creatinine present in the body is in the phosphorylated form as phosphocreatine in the skeletal muscles 32. Synthesis of creatine mainly takes place in the kidney, pancreas, and liver; the phosphorylated forms act as an instant source of energy and the increase in creatine is associated with energy demand 33. Phosphocreatine plays a crucial role in cellular energy homeostasis by keeping a fair balance between ATP and ADP during intense periods of energy demand. Creatine acts as a cellular buffer by utilizing ADP and H+ ions produced during hydrolysis of ATP and from accumulating lactate. Thus, reduces the effects of muscle fatigue by creating an optimum level of pH and also regulates increased levels of ADP from inhibiting ATP functions. The decreased levels of creatine/creatinine in CS animals might be related to the decrease in muscle mass resulting from stress induced weight loss Supplementary Figure S2.

N-acetyl glycoproteins (NAG) and O-acetyl glycoproteins (OAG) are acute-phase reaction glycoproteins detected in the rat serum. The O-acetylated carbohydrate-bound protein resonance present in rat serum, are surrogate “acute-phase” glycoproteins in animal models of human inflammatory conditions 34. NAG and OAG are acute phase proteins, acting as inflammatory intermediaries and could be a response to tissue damage, and thus, the increased concentrations of serum NAG and OAG in AS groups were likely to reflect an inflammatory response resulting from the restrained stress procedure. Elevated levels of N-acetyl glycoproteins and O-acetyl glycoproteins in blood serum of oxidative stressed animals are consistent with previous investigations of the metabolic response to stress 35. However, the NAG and OAG, were found to be depleted in the CS groups which might be associated with the decreased liver efficiency in synthesis of these glycoproteins 36 may be responsible for these lower levels of -acetyl signal in the CS serum.

## Conclusion

This study paradigm uses restrained stress on Sprague-Dawley rats, using ^1^H NMR spectroscopy coupled with multivariate analysis to explore the metabolic profiles in serum of Acute and chronic stress models. CS group exhibited slight but significant change in behavioural response while AS group exhibited highly increased level of anxiogenic behaviour with respect to the control group. On the basis of behavioural outcomes several metabolites were found to be specifically modified. The altered metabolic entities include neurotransmitter, energy metabolism, membrane components and amino acids, which may be the outcome of the adaptive measures taken by the body in response to stress conditions. These findings provide a new insight into metabolic changes induced under restrained stress and the underlying mechanism**.**

## Nanotheranostics approach and Future direction

Theranostics is made up of two words- Therapeutics and Diagnostics which means the diagnosis and treatment of the disorder. Whereas Nanotheranostics means the treatment of the disorder by using the nanotechnology. Nanotechnology means the use of nanoparticles in the treatment of disorder. NMR technique is a powerful tool to evaluate the drugs and can help in the development of new agents in order to provide more beneficial effects. Stress is a factor playing a very crucial role in the alteration of behaviour and mood or psychiatric disorder. By using the NMR apporach, altered metabolites in acute or chronic stress serum samples can be investigated and here the role of nanotheranostics comes into play. By designing the nanoparticles that can target their specific biomarkers and can allows us the real time monitoring of the particular disorder.

For the future direction, on the basis of present study, it will be interesting to further investigate which particular neuroanatomical regions are involved in such anxiety-like behaviour and metabolic changes in that particular region and whether the epigenetic alterations also contribute to them. Metabolites associated with these particular changes may also help in revealing a more specific target for procurement of the concerned disorder.

## Supplementary Material

Supplementary figures.

## Figures and Tables

**Table 1:** Details of the metabolites best describing the variation between Acute and Chronic -stress groups with respect to Control group

S.No	Metabolite	ppm	Acute Stress			Chronic Stress		
			VIP	Level ↑/↓	AUROC	VIP	Level ↑/↓	AUROC
*1*	HDL	0.81-0.83	2.1	↑	0.96	2.6	↑	0.96
*2*	Isoleucine	0.92	1.6*	↑	0.96	≠	≠	≠
*3*	Leucine	0.94	1.8	↑	0.92	≠	≠	≠
*4*	Valine	1.03	1.3*	↑	0.92	≠	≠	≠
*5*	3-HB	1.20	1.3	↑	0.92	1.58	↓^#^	0.76
*6*	V/LDL	1.21-1.24	1.8	↓	0.92	2.7	↑	0.84
*7*	Lactate	1.31	2.5	↑^#^	0.64	6.6	↓	0.88
*8*	Alanine	1.46	1.1	↓^#^	0.72	3.1	↓	1
*9*	Citrulline	1.88	1.0	↑	0.88	1.4	↓	1
*10*	Acetate	1.91	1.2	↑^#^	0.84	1.8	↓	0.92
*11*	NAG	2.03	2.3	↑^#^	0.88	1.4*	↓^#^	0.72
*12*	Glutamate	2.07	1.1*	↑	0.88	1.2	↓	0.84
*13*	OAG	2.12	2.4	↑	0.88	2.2	↓^#^	0.84
*14*	Pyruvate	2.36	1.3	↑	0.84	≠	≠	≠
*15*	Succinate	2.39	1.3	↓^#^	0.8	3.7	↓	0.88
*16*	Glutamine	2.43	1.1	↑	0.84	1.1*	↓	0.76
*17*	Creatin -e/ine	3.00	1.1	↑	0.76	1.3	↓	0.84
*18*	Choline/PC	3.18	1.5*	↑	0.96	1.2*	↓	0.84
*19*	TMAO	3.25	≠	≠	≠	2.5	↑^#^	0.72
*20*	Glucose	3.39	4.1	↓	0.84	4.0	↑	0.88
*21*	Acetoacetate	3.43	1.0*	↑	0.88	1.6	↓	1
*22*	Glycine	3.54	≠	≠	≠	1.7	↑^#^	0.76
*23*	Myo-Inositol	3.59	1.2	↑	0.92	≠	≠	≠
*24*	Glycerol	3.63	1.4	↑	0.88	1.4	↓	0.92
*25*	Lysine	3.74	1.0	↑^#^	0.76	2.5	↓	0.96
*26*	Serine	3.94	1.1	↑	0.76	1.4	↓	1
*27*	PUFA	5.26	1.1	↑	0.92	1.7	↑	0.96
*28*	Tyrosine	6.87	1.2*	↑	0.76	1.8	↓	0.92

The up and down arrows represent an increase or decrease in metabolite levels responsible for class separation with significant changes.

## Abbreviations

NMR: Nuclear Magnetic Resonance
CPMG: Carr-Purcell-Meiboom-Gill
AUROC: Area under the ROC curve
PCA: Principal Component Analysis
PLS-DA: Partial Least Squares Discriminant Analysis
RS: Restraint stress
AS: Acute stress
CS: Chronic stress
NC: Normal control
