# Supplementary material for: Pre-clinical Investigations of Therapeutic Markers Associated with Acute and Chronic Restraint Stress: A Nuclear Magnetic Resonance Based Contrast Metabolic Approach
Source: Nanotheranostics. 2023 Jan 1;7(1):91–101. doi: 10.7150/ntno.76294 (PMC9760362; doi:10.7150/ntno.76294)
Supplement: Supplementary file 1 — Supplementary figures. [file ntnov07p0091s1.pdf]

Contrasting Metabolic Patterns Associated with Acute and Chronic Restraint Stress in Sprague Dawley Rats: A ^{1}H-NMR-Based Metabolomics Study

Sanjay Singh[1], Sukanya Tripathy[1], Atul Rawat[1,2], Durgesh Dubey[1,2], Sarfraj Ahmad Siddiqui[1], Rajesh Ugale[4], Dinesh Kumar[2], Anand Prakash[3]*

[1]Department of Biotechnology, Babasaheb Bhimrao Ambedkar University, Lucknow, India

[2]Centre of Biomedical Research, Lucknow, India

[3]Department of Biotechnology, Mahatma Gandhi Central University, Bihar, India

[4]Department of Pharmaceutical Sciences, Rashtrasant Tukadoji Maharaj Nagpur University, Nagpur, India

*** Author for Correspondence**

Anand Prakash: anandprakash@mgcub.ac.in

KEYWORDS: Metabolomics, NMR, Metabolic Patterns, Restrained Stress, Behavioural Metabolomics.

ABBREVIATIONS: NMR-Nuclear Magnetic Resonance; CPMG-Carr–Purcell–Meiboom–Gill; AUROC-Area under the ROC curve; PCA-Principal Component Analysis; PLS-DA-Partial Least Squares Discriminant Analysis; RS-Restraint stress; AS-Acute stress; CS-Chronic stress.

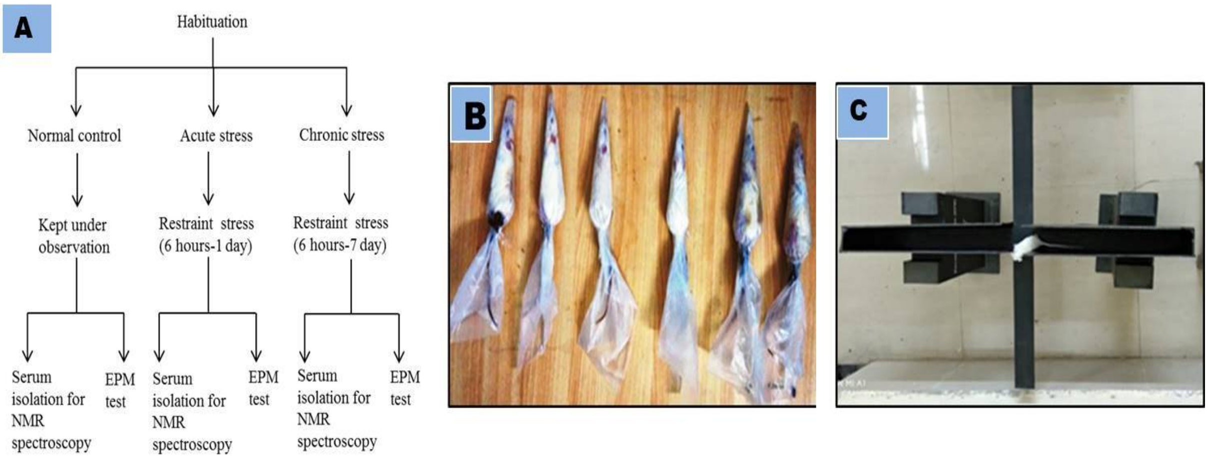

Figure S1: (**A**) represents the protocol used in the study. (B) represents the restraint stress given in plastic cone. (**C**) animals were tested on the EPM to know the effect of stress duration on the anxiety.

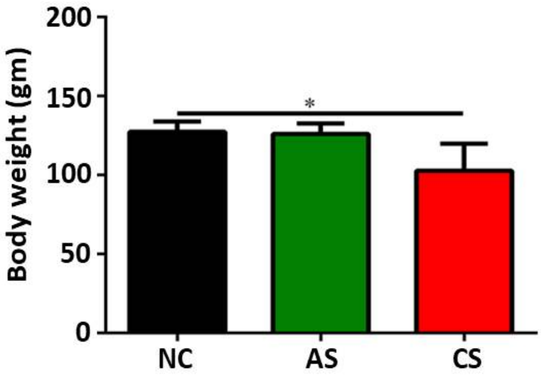

Figure S2: represents the effect of AS and CS on the body weight of animals. CS grouped animals showed significant decreased body weight ($p<0.05$) as compared to the NC and AS group while no change was observed in between the NC and AS grouped animals ($p>0.05$).

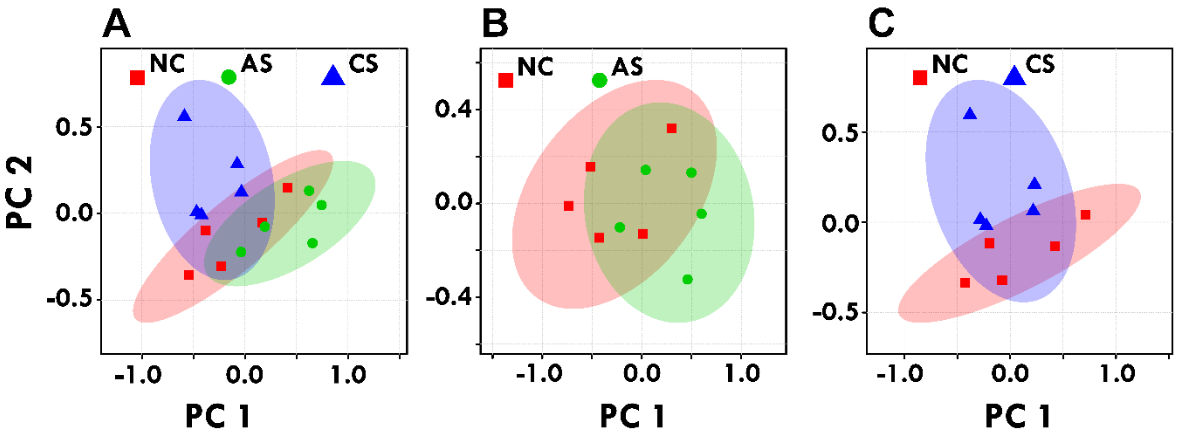

Figure S3: (A) Combined 2D PCA score plot comprising of all 3 groups NC, AS and CS. (B) and (C) Pairwise 2D PCA score plot of AS and CS with the NC respectively. Derived from 1D ^{1}H NMR CPMG spectra of serum samples.

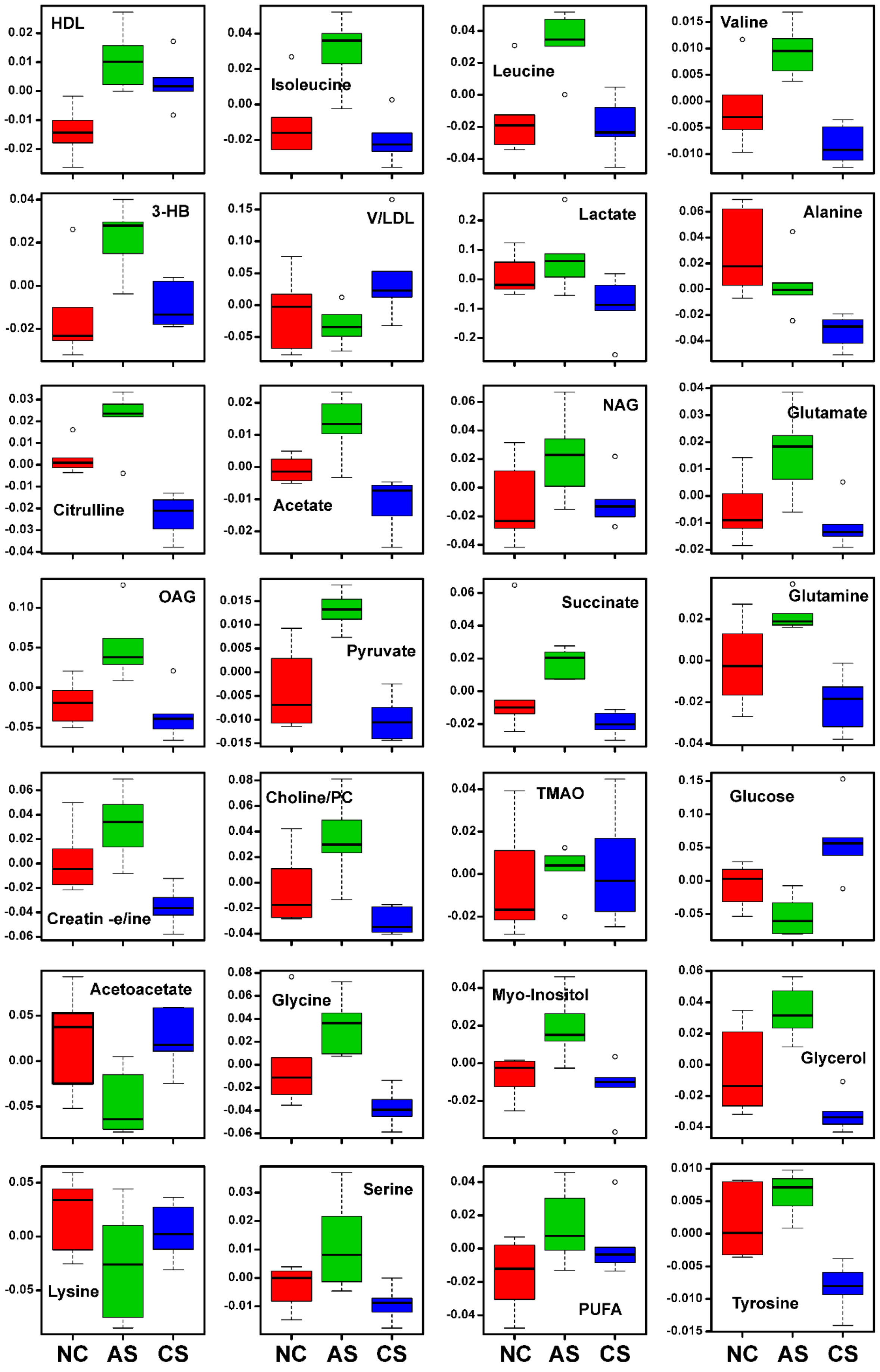

Figure S4: Box-whisker plots of metabolites that were significantly perturbed in AS and CS with respect to the Control group derived from 1H NMR CPMG spectra.

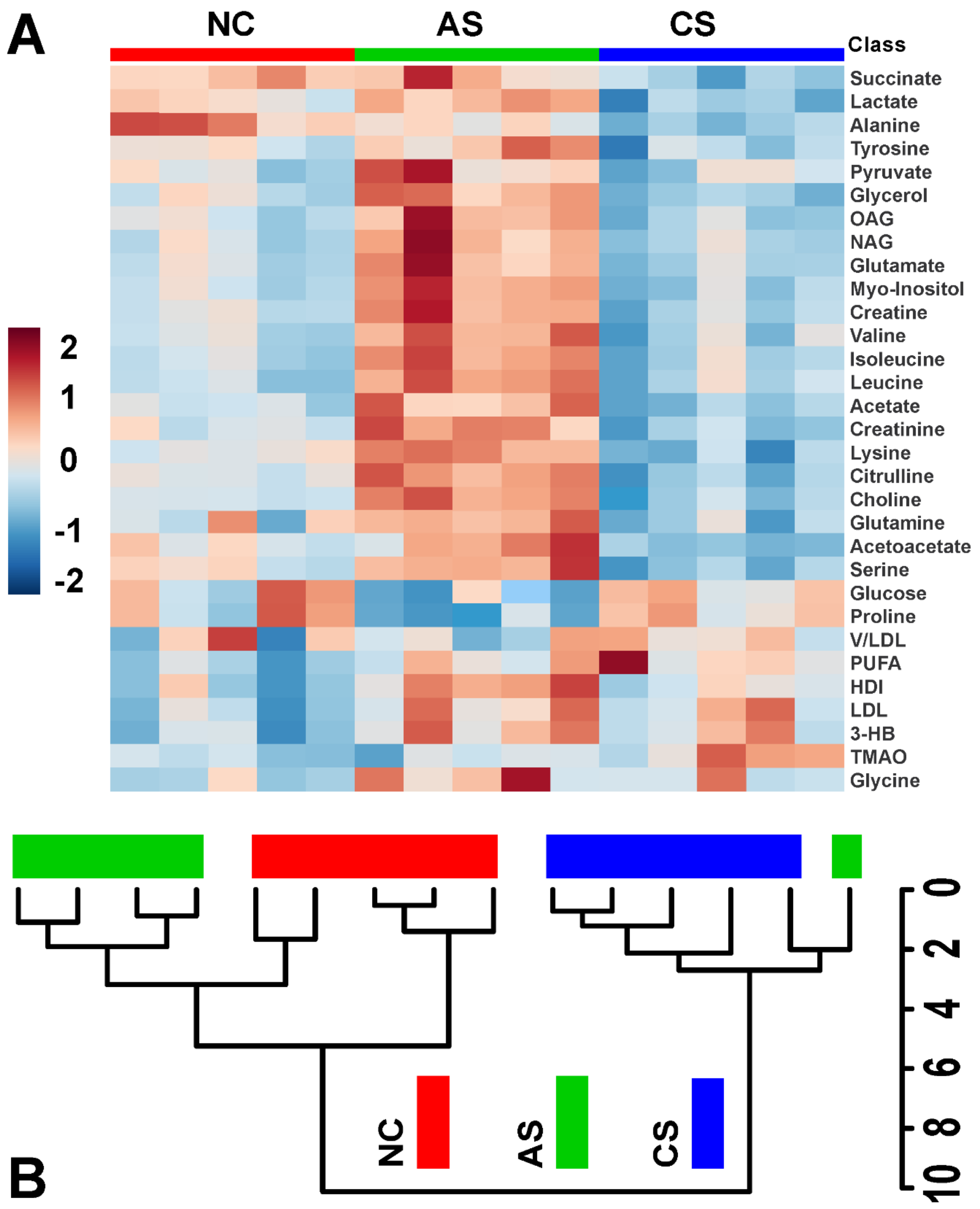

Figure S5: (A) Heat map of statistically significant metabolites in between Acute and Chronic -stress groups with respect to Control group as derived from CPMG 1H NMR Spectra. Red=elevated; Cyan=reduced. Each column represents a sample, and each row represents a metabolite. (B) Hierarchical Clustering Analysis The dendrogram of control, acute and chronic –stress groups.
